# Preactivation Crosslinking—An Efficient Method for the Oriented Immobilization of Antibodies

**DOI:** 10.3390/mps2020035

**Published:** 2019-05-03

**Authors:** Barbara Schroeder, Hoa Le Xuan, Jule L. Völzke, Michael G. Weller

**Affiliations:** 1 Federal Institute for Materials Research and Testing (BAM), Division 1.5 Protein Analysis, Richard-Willstätter-Strasse 11, 12489 Berlin, Germany; barbara.schroeder@fu-berlin.de (B.S.); le.hoa@web.de (H.L.X.); jule.voelzke@bam.de (J.L.V.); 2 Institute of Pharmacy, Medicinal Chemistry, Freie Universität Berlin, Königin-Luise-Strasse 2+4, 14195 Berlin, Germany

**Keywords:** antibody coating, proximity-enhanced reaction, immunoglobulins, IgG, Protein A, Protein G, bio-interaction, immunoprecipitation, pull-down assay, immunocapture, stabilization, yield, regeneration, nanoparticles, microparticles, biochips, immunosensor, photochemical crosslinker, click chemistry, Herceptin, Trastuzumab

## Abstract

Crosslinking of proteins for their irreversible immobilization on surfaces is a proven and popular method. However, many protocols lead to random orientation and the formation of undefined or even inactive by-products. Most concepts to obtain a more targeted conjugation or immobilization requires the recombinant modification of at least one binding partner, which is often impractical or prohibitively expensive. Here a novel method is presented, which is based on the chemical preactivation of Protein A or G with selected conventional crosslinkers. In a second step, the antibody is added, which is subsequently crosslinked in the Fc part. This leads to an oriented and covalent immobilization of the immunoglobulin with a very high yield. Protocols for Protein A and Protein G with murine and human IgG are presented. This method may be useful for the preparation of columns for affinity chromatography, immunoprecipitation, antibodies conjugated to magnetic particles, permanent and oriented immobilization of antibodies in biosensor systems, microarrays, microtitration plates or any other system, where the loss of antibodies needs to be avoided, and maximum binding capacity is desired. This method is directly applicable even to antibodies in crude cell culture supernatants, raw sera or protein-stabilized antibody preparations without any purification nor enrichment of the IgG. This new method delivered much higher signals as a traditional method and, hence, seems to be preferable in many applications.

## 1. Introduction

Antibodies are one of the most important biochemical reagents. They can be used in immunoassays [1,2], biosensors [3,4,5,6,7], microarrays [8,9], atomic force microscopy [10], surface plasmon resonance [11,12], affinity chromatography [13,14], affinity purification-mass spectrometry [15], mass spectrometric immunoassay [16], immunoprecipitation [17], and magnetic particle separation [18] for the application in diagnostics, food and environmental analysis, medical and biochemical research. Many of these techniques require the immobilization of the respective antibody to a surface. Although the random attachment of the immunoreagent is common due to its simplicity, oriented immobilization is usually considered to be preferable [12,19,20,21,22,23]. A multitude of techniques has been proposed for the oriented immobilization of antibodies. However, only the use of secondary antibodies, (strept)avidin, Protein A [24] or G [25] and the periodate method [26] have been used more frequently. In some cases, the reversibility of such complexes is seen as an advantage since the surface can be regenerated by the release of the primary binding reagent. However, for preparative applications or sample preparation for mass spectrometry (e.g., immunocaptureLC-MS/MS), the elution of the immunoreagent leads to unwanted contamination of the sample or product. Besides, the expensive antibody may be lost during the elution step. In these cases, either non-oriented covalent techniques are used, or the oriented Protein A/G/antibody complex needs to be stabilized with crosslinking reagents. Unfortunately, with conventional crosslinkers, a targeted approach is challenging, which leads to the random derivatization of many antibody side chains and amino-termini. Since crosslinkers have been used heavily for the examination of protein–protein interactions in general, these reactions have been studied in some detail. However, up to now, the random-derivatization characteristics were accepted as an inevitable consequence of this approach. It must be noted that the N-termini of antibodies are quite near to their binding sites, which makes a potentially negative influence of amino-reactive reagents quite likely. Since the variable region of antibodies shows individual structures and properties, the prediction of such problems, e.g., the loss of binding capacity, is nearly impossible today.

**Figure 1:**
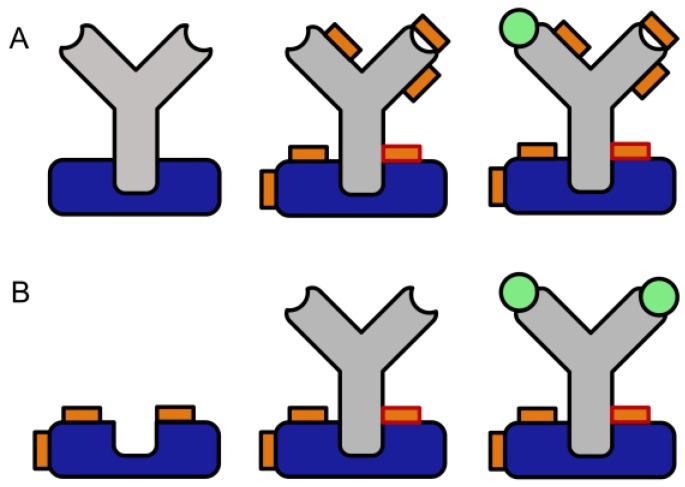
Comparison of conventional crosslinking (**A**) to the proposed preactivation crosslinking method (**B**). Please note the potentially higher binding capacity of the immobilized antibody and the complete lack of chemical modification in the F_ab_ region (blue: Protein A or G, grey: antibody, orange: crosslinker, orange with red rim: protein-protein crosslink, orange with dark rim: intramolecular or half crosslink, green: antigen).

To overcome these limitations, we developed a novel two-step crosslinking method (Figure 1). In these protocols, the antibody capturing molecule is pre-activated with “slow” crosslinkers, and subsequently, any residual reagent is washed away to avoid any contact of the free crosslinking reagent with the antibody. “Slow” in this context means the property that in a bifunctional crosslinker, the first reaction does not lead to the hydrolysis or otherwise deactivation of the second function. This concept shows some similarity with photochemical crosslinking [27], which has been used in the exploration of nearly all types of bio-interactions. However, photochemical linkers have some significant disadvantages, which may have limited their more widespread application. The most obvious drawback is their light sensitivity, which requires appropriate countermeasures during synthesis, purification, and use. Accidental exposure to light might reduce the conjugation yield in an irreproducible way. Furthermore, the reaction yields of photochemical reactions often are low [27]. Also, the required setup for UV irradiation adds complexity to the experiments, the progression of the reaction is difficult to monitor, and unwanted photochemical byproducts may be formed. Some short wavelength lamps also need additional safety measures to avoid unwanted exposure of the laboratory workers. Finally, the possibility of the direct introduction of a photo-inducible group in a recombinant protein [28], leads to a complicated and expensive production, which might preclude commercial availability even in the future.

Our work is also related to the concept of heterofunctional supports in the field of enzyme immobilization, which has been presented and discussed in several papers [29,30,31,32].

One of the most popular applications for which chemical crosslinking plays an important role is the immobilization of antibodies on magnetic or other beads. Particles pre-coated with Protein A or G are readily available from many commercial suppliers. Most of the protocols delivered by the manufacturer suggest crosslinking the Protein G/IgG complex by use of chemical crosslinkers, such as bis(sulfosuccinimidyl)suberate (BS3), to avoid co-elution of the antibody. However, the formation of many byproducts and the potential inactivation of the antibodies is rarely considered at all.

In recent years, some quite smart concepts have been presented, to achieve “proximity-enhanced” or “proximity-enabled” crosslinking reactions in biochemical complexes. Xiang et al. [33,34,35,36] showed the introduction of haloalkane-modified tyrosine residues for this purpose. Very recently, a similar concept was published based on haloalkane-modified lysines [37]. Furthermore, lysines modified with a fluoroacetamide group were used in combination with a cysteine to introduce defined crosslinks in proteins or protein complexes [38]. In addition, Furman et al. [39] and Xuan et al. [40] presented other reactive groups for the same purpose. All of them require the site-specific introduction of artificial amino acids [41,42], e.g., by tRNA-synthetases. This limits the applicability to genetically modified proteins [43] and may be the reason for their lack of practical use. In contrast, our approach can be used for any protein or peptide, irrespective of their source, if a favorable (bio-) interaction can be formed.

## 2. Materials and Methods

### 2.1. Chemicals and Reagents

Laboratory water was obtained from a Milli-Q water purification system (Millipore, Bedford, MA, USA). Dimethylsulfoxide (DMSO), was from AppliChem (Darmstadt, Germany), bovine serum albumin (BSA), disodium hydrogen phosphate dihydrate, sodium dihydrogen phosphate dihydrate, sodium chloride, citric acid monohydrate, and trisodium citrate dihydrate, sodium cyanoborohydride, sulfuric acid, Tween 20, sodium tetraborate decahydrate were obtained from Sigma-Aldrich (Steinheim, Germany). The reagents for crosslinking were obtained from the following sources: Succinimidyl iodoacetate (SIA), bis(sulfosuccinimidyl) suberate, 1-ethyl-3-(3-dimethylaminopropyl) carbodiimide hydrochloride (EDC), N-hydroxysuccinimide (NHS) were obtained from Thermo Scientific, succinimidyl(4-iodoacetyl) aminobenzoate) (SIAB) and sulfosuccinimidyl (4-iodoacetyl) aminobenzoate) (Sulfo-SIAB) were obtained from Apollo Scientific (Bredbury, UK). Glutaraldehyde, 1,3-butadiendiepoxide and formaldehyde were obtained from Sigma-Aldrich (Steinheim, Germany), disuccinimidyl tartrate was from CovaChem, glyoxal was from Merck (Darmstadt, Germany) and tris(hydroxymethyl)phosphine was bought from abcr (Karlsruhe, Germany). Protein G (pro-402-c) and recombinant Protein A (pro-774) were purchased from ProSpec (Ness-Ziona, Israel). The microtitration plates were from Greiner bio-one (Frickenhausen, Germany). Mouse Monoclonal antibodies to horseradish peroxidase (HRP), clone HP-03 (IgG1), product No. 11-262-C100 were obtained from EXBIO (Praha, Czech Republic). The humanized antibody Herceptin (Trastuzumab) was kindly supplied by Roche (Penzberg, Germany). In this article, Herceptin is referred to as “human IgG_1_”, due to its human Fc domain. It was purified from any additives by Protein A chromatography. TMB substrate was obtained from Seramun GmbH, Heidesee, Germany.

### 2.2. Buffers

Phosphate-buffered saline (PBS), pH 7.4: 2.3 mM KH_2_PO_4_, 10 mM Na_2_HPO_4_*2 H_2_O, 136.9 mM NaCl, Phosphate buffer, pH 6: 12.3 mM Na_2_HPO_4_*2H_2_0, 61.0 mM NaH_2_PO_4_*2 H_2_O, Phosphate buffer, pH 7: 87.7 mM Na_2_HPO_4_*2H_2_0, 39.0 mM NaH_2_PO_4_*2 H_2_O, Sodium borate buffer (SB), pH 8 and 9: 10.0 mM Na_2_B_4_O_7_*10 H_2_O, Citrate buffer, pH 5: 35.0 mM Citric Acid*H_2_O, 65.0 mM Trisodium citrate*H_2_O, Washing buffer (PBS-Tween), pH 7.4: pH 7.4: 1.3 mM KH_2_PO_4_, 6.6 mM Na_2_HPO_4_*2 H_2_O, 0.5 mM Tween 20, Elution buffer (Glycine/HCl), pH 2.3: 0.1 M Glycine, titrated to pH 2.3 with 0.1 N HCl.

### 2.3. Equipment

ELISA washer: 405 Select BioTek, ELISA reader: EPOCH 2 BioTek, multichannel pipettes: Eppendorf Xplorer plus, Brand Transferpipette S, balance Mettler Toledo XS105 Dual Range, centrifuge Hettich Mikro 220R, UV VIS spectrophotometer ThermoScientific Evolution 220.

### 2.4. Crosslinking Protocol with SIAB or Sulfo-SIAB

**Figure 2:**
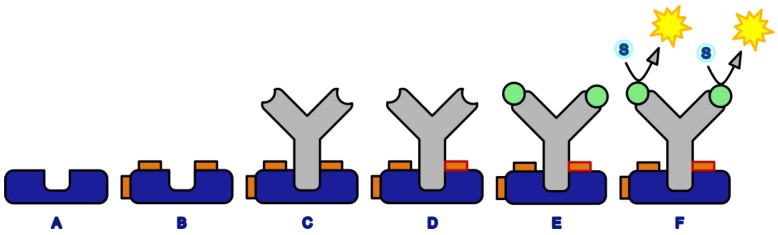
Crosslinking assay for the screening of potential crosslinkers (blue: Protein A or G, grey: anti-horseradish peroxidase antibody, orange: crosslinker, orange with red rim: protein-protein crosslink, orange with black rim: intramolecular or half crosslink, green: horseradish peroxidase (antigen), yellow: chromogenic product. (**A**) Coating with Protein G, (**B**) Preactivation with the crosslinker, (**C**) Antibody binding to Protein G, (**D**) Formation of crosslink, (**E**) Binding of antigen (enzyme), (**F**) Enzymatic formation of chromogenic product (washing steps are not shown).

Crosslinking assays (Figure 2) were performed in 96-well polystyrene microtitration plates (MTP). Protein G was diluted in phosphate-buffered saline (PBS, pH 7.4) to a final concentration of 10 mg/L. Then, 100 µL of this solution was pipetted into each well of the MTP, which was shaken for at least 90 min at 750 rpm. The plate was washed three times with PBS-Tween. In the next step, 300 µL of a 1% solution of bovine serum albumin (BSA) was added to each well. This blocking step was performed for at least one hour at a shaking frequency of 750 rpm. The plate was subsequently washed three times. Then 100 µL of the crosslinker solution was added to the wells. Suitable concentrations have been determined as follows: 0.25 mM for SIAB and 1 mM for sulfo-SIAB. For poorly soluble crosslinkers, such as SIAB, DMSO can be used as solubilizer with a subsequent dilution in a buffer to a maximum final concentration of 40% of solvent. After a reaction time of 15 min, residual crosslinker was removed by three washing steps. The monoclonal anti-peroxidase antibody was diluted 1:100,000 (10 ng/L) in phosphate buffer (pH 6) and added to the wells (100 µL per cavity). This solution was incubated at 750 rpm for sixteen hours and removed by three washing steps with PBS-Tween. A solution of 10 mg/L of horseradish peroxidase was prepared in PBS-Tween-BSA as described above. Then, 100 µL was added to each well and shaken at 750 rpm for 15 min. Subsequently, the plate was washed three times with PBS-Tween. Finally, 100 µL per well of TMB substrate was added and incubated for 1–30 min, as required to reach a sufficient absorbance. After this development time, the reaction was stopped with 0.25 M sulfuric acid. The absorbances were recorded with a microplate reader at 450 nm (650 nm reference wavelength).

### 2.5. Crosslinking Protocol with Glutaraldehyde

First, the protocol was performed as described in Section 2.4 with Protein G. Instead of SIAB solution, 100 µL of glutaraldehyde (2 mM) dissolved in sodium borate buffer (pH 8) was added to the wells. After a reaction time of 15 min, residual crosslinker was removed by three washing steps. The monoclonal anti-peroxidase antibody was diluted 1:100,000 (10 ng/L) in phosphate-buffer (pH 6) with 0.1% of Tween 20 and 1% of bovine serum albumin (BSA) and added to the wells (100 µL per cavity). This solution was incubated at 750 rpm for one hour and removed by three washing steps with PBS-Tween. In the following step, 100 µl of NaCNBH_3_ (200 µg/mL) in PBS was added to the wells to reduce imines to stable secondary amines. A one-hour incubation was required for the reduction (750 rpm), and subsequently, the solution was removed by three washing steps with PBS-Tween. The rest of the protocol followed the steps described in Section 2.4.

### 2.6. Comparison of Crosslinking Protocols

The experiment was performed in analogy to the protocol 2.4. 100 µL of a Protein G solution (10 mg/L, PBS pH 7.4) was pipetted to a microtitration plate and incubated for 90 min at room temperature under shaking. After a washing step (PBS-Tween, pH 7.4), blocking was performed with 300 µL of BSA solution (PBS pH 7.4) for one hour. After another washing step, 100 µL of sulfo-SIAB (100 µL, 1 mM, PBS, pH 7.4) was incubated for 30 min. The wells for the BS3 crosslinker were supplied with 100 µL of PBS. After a subsequent washing step, the whole plate (except controls) was incubated with a murine monoclonal antibody (HP-03, IgG1, 100 µL, 1:100,000, phosphate buffer, pH 6.0) for 16 h. After the next washing step, the sulfo-SIAB wells were supplied with 100 µL of PBS, and the BS3 wells were incubated with 100 µL of BS3 in PBS (pH 7.4) for 30 min under shaking. After the next washing step, any non-crosslinked antibody was removed with elution buffer (pH 2.3, glycine/HCl, 30 min) under shaking. After a further washing step, the plate was supplied with 100 µL of horseradish peroxidase (HRP, 10 mg/L, PBS-Tween + 1% BSA) and incubated for 15 min. After a final washing step, the 100 µL of TMB substrate was added, incubated for 10 min and stopped with diluted sulfuric acid. The signal was recorded at 450 nm. A blank value (without crosslinker) was subtracted.

## 3. Results

### 3.1. Crosslinking Assay Based on Protein G–Mouse IgG Interaction

Well-known protein interaction pairs were chosen for this study. Recombinant Protein G, a protein from *Streptococcus*, (or Protein A), and a murine monoclonal antibody (IgG_1_) against horseradish peroxidase (HRP) were used. The advantage of the latter is its antigen, which can be easily determined in a microtitration plate (MTP) format and hence is ideally suited for screening purposes. Protein G (or A) were adsorbed to the MTP, washed and subsequently activated with the respective crosslinker. Any excess of the reagents was easily removed by washing steps; this is a big plus of any heterogeneous format. Furthermore, this setup simplifies any pH variation by complete buffer exchange. After the activation step, the antibody was added in a suitable conjugation buffer. After a suitable conjugation time, the conjugation yield was determined by elution of the antibody by acidic buffer (glycine/HCl). Any non-conjugated antibody will be released; the conjugated fraction will stay immobilized on the plate surface. In the next step, horseradish peroxidase was added and incubated. After the next washing step, a chromogenic substrate, based on tetramethylbenzidine, and hydrogen peroxide were added. After a suitable development time, the color reaction was stopped by acid and absorbance was measured with an MTP-reader. This assay (Figure 2) was designed for the convenient examination of the preactivation crosslinking procedure.

### 3.2. Crosslinker Screening

A considerable number of crosslinkers have been proposed, and quite a few of them are commercially available. The most frequently used ones seem to be based on N-hydroxysuccinimide (NHS) chemistry or their sulfo-derivatives [44,45,46], targeted against the ε-amino group of the lysine side chain and the N-terminus of a peptide or protein. We have chosen a series of crosslinkers based on different chemistries for a prescreening. There are several criteria, which are relevant for the selection of a suitable crosslinker, for example, the chemical reactivity, the linker length and flexibility, the hydrophobicity, the solubility and stability of the reactive groups in aqueous buffers, their pH preference and many more. In Table 1, a list of crosslinkers is shown, which have been used for the screening.

**Figure 3:**
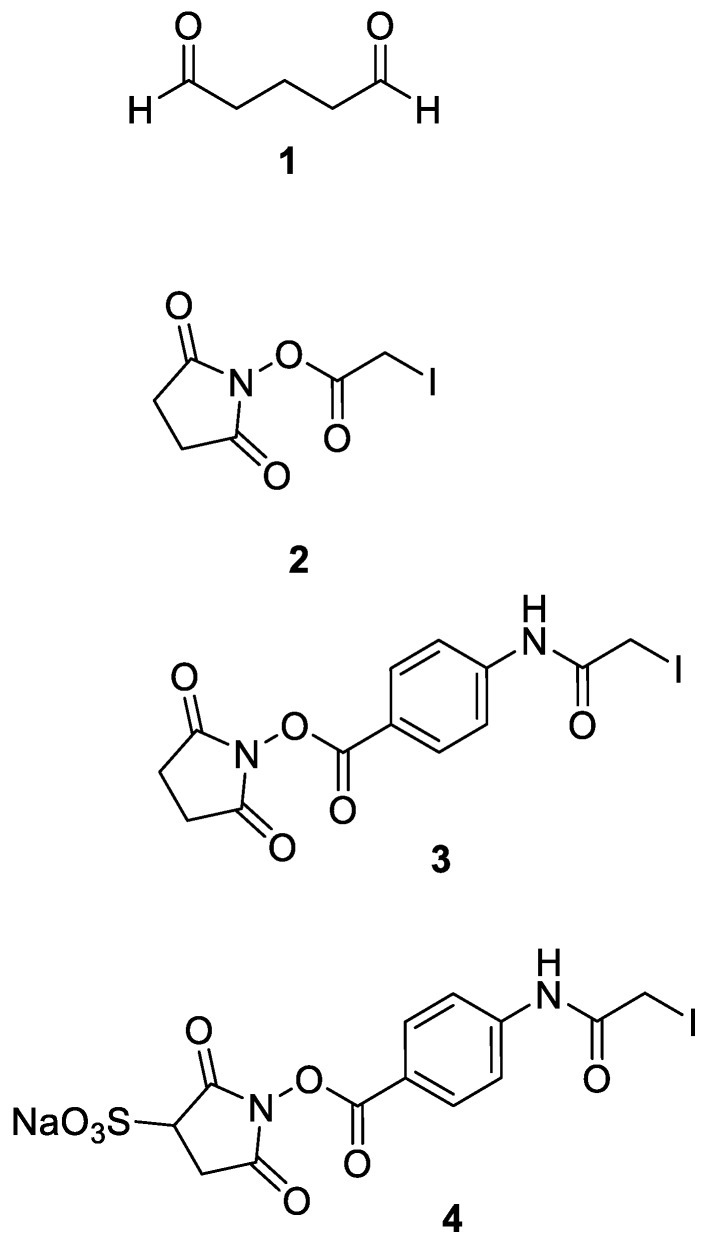
Chemical structures of crosslinkers, which have been found particularly suitable for preactivation protocols: Glutaraldehyde (**1**), Succinimidyl iodoacetate (**2**), Succinimidyl(4-iodoacetyl) aminobenzoate (**3**), Sulfosuccinimidyl(4-iodoacetyl)aminobenzoate (**4**).

In Table 1, the results are summarized as – to +++, where – stands for no crosslinking and +++ denotes a very high signal caused by crosslinked Protein G/antibody complex. It has to be noted that many crosslinkers, which are perfectly suitable for normal crosslinking protocols (reaction with the preformed complex), such as BS3, are not or only weakly active in the new format. In the novel preactivation format only a few crosslinkers have proven to be suitable: Particularly glutaraldehyde (GA) [50,55,56], succinimidyl iodoacetate (SIA) [53], sulfosuccinimidyl iodoacetate (sulfo-SIA), succinimidyl (4-iodoacetyl)aminobenzoate (SIAB) [48] and sulfosuccinimidyl (4-iodoacetyl)aminobenzoate (sulfo-SIAB) [54] can be recommended. All further experiments have been focused on these pre-selected reagents (Figure 3).

### 3.3. Influence of pH on the Crosslinking of Protein G with Murine IgG_1_

**Figure 4:**
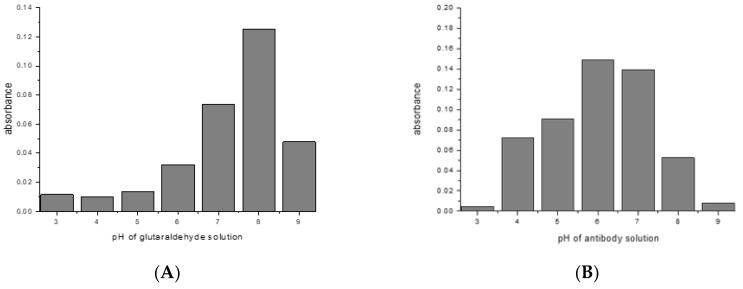
Glutaraldehyde activation of Protein G: (**A**) pH influence of crosslinker solution (**B**) pH influence of antibody solution (mouse IgG_1_) in the same experiment.

Most crosslinking reactions are highly pH dependent. This was explored with the examples of glutaraldehyde/mouse IgG1/Protein G, and SIAB/mouse IgG1/Protein G. For glutaraldehyde, it could be shown that a pH of 8 seems to be optimal for preactivation (Figure 4A). This means that a standard buffer, such as PBS of a pH 7.4 is a suitable option. In the second step, the addition of the mouse IgG1, a pH of 6 seems to be preferable (Figure 4B). This might be dominated by the binding optimum of the murine IgG1/Protein G pair, which has been determined as pH 4–6 [57,58]. In addition, it was observed that increasing the incubation time of the glutaraldehyde leads to increasing immobilization yields (data not shown).

**Figure 5:**
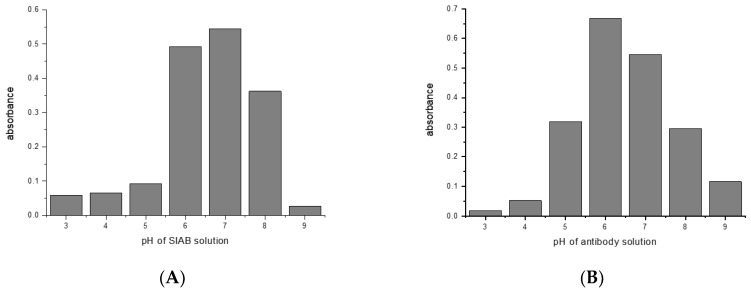
SIAB activation of Protein G: (**A**) pH influence of crosslinker solution (**B**) pH influence of antibody solution (mouse IgG_1_) in the same experiment.

For SIAB a pH around 7 was found to be optimal for preactivation (Figure 5A). This also means that a standard buffer, such as PBS pH 7.4 might be a good option. Similar to the situation with glutaraldehyde, a pH of 6 was found to be preferable (Figure 5B) for the antibody (mouse IgG1). In experiments with Protein A, a preferable pH of 7.4 was found (not shown), in accordance with the reported IgG/Protein A optimum. This also supports the notion that not the linker, but the protein–protein interaction governs the second step. This has to be taken into consideration when new crosslinking pairs should be explored.

**Figure 6:**
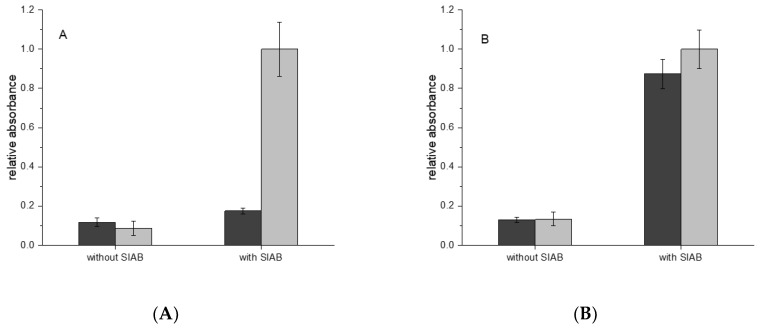
(**A**) Mouse IgG_1_: Preactivation immobilization with SIAB on Protein A (dark grey) or Protein G (light grey); (**B**) Human IgG: Preactivation immobilization with SIAB on Protein A (dark grey) or Protein G (light grey).

In the next step, the sequential crosslinking with the systems Protein A and Protein G in combination with murine IgG_1_ and human IgG was examined. In the case of IgG_1_ from mouse, a monoclonal antibody against horseradish peroxidase was used as a model system. In Figure 6A it could be shown that Protein A/IgG_1_ leads to a much lower signal than Protein G/IgG_1_. In the case of human IgG, Protein A and Protein G lead to very similar immobilization results (Figure 6B).

The species specificity of Protein A, G, and other IgG binding molecules had been explored in detail [59]. Hence, it is well-known that mouse IgG_1_ binds only weakly to Protein A, in contrast to human IgG, which is a strong binder. These properties could be confirmed in our system. This is also clear support of the selectivity of this crosslinking procedure. If the crosslinker alone would be responsible for the immobilization, no such behavior would be expected. Furthermore, the addition of BSA to the antibodies did not influence the immobilization significantly (data not shown). This also substantiates the highly selective mechanism and contradicts any simple protein crosslinking hypothesis. In this case, any presence of any irrelevant protein should heavily compete with the desired immobilization process, which is a frequent problem in conventional immobilization procedures.

### 3.4. Incubation Time of SIAB-Activated Protein G with Mouse IgG_1_

**Figure 7:**
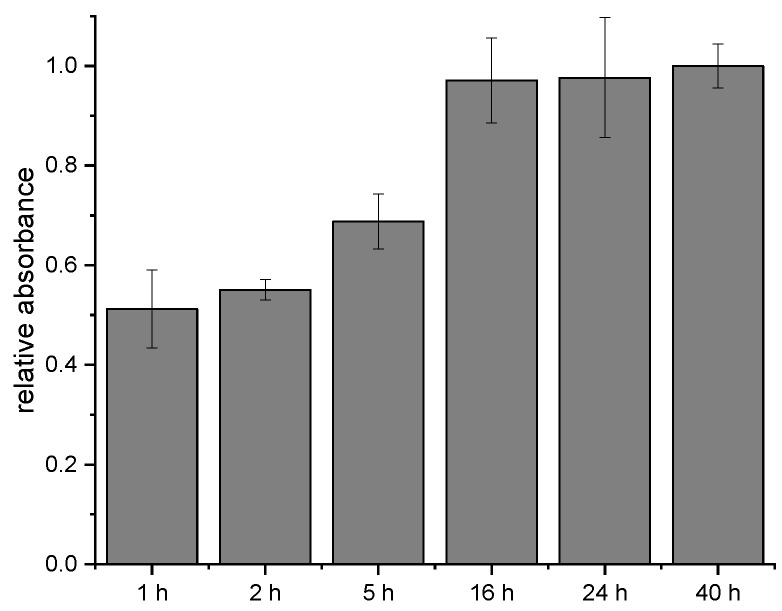
Influence of the incubation time on the SIAB-based preactivation immobilization of mouse IgG_1_ on Protein G.

In the next experiments, the time-dependency of the crosslinking process with SIAB was explored. It could be shown that some of the crosslinkers seem to bind relatively fast, in contrast to others, which need several hours to reach a maximum signal. After one hour of antibody incubation, about 50% of the maximum was achieved already. After 16 h, the signal doubled. Further extension of the incubation time did not increase the response anymore. The non-linear increase indicates that at least two different rate constants, and hence two different crosslinking sites may be involved. In general, 24 h should be more than sufficient to reach a maximal signal (Figure 7).

### 3.5. Influence of Solvents on the Crosslinking Process

The water solubility of different crosslinkers varies widely. Particularly, SIAB is poorly water soluble. Hence, concentrated SIAB solutions cannot be prepared in the usual buffers. Hence, SIAB should be pre-dissolved in organic solvents such as methanol or DMSO. Interestingly, we found that DMSO leads to more efficient activation of Protein G than methanol. With 40% of DMSO, a concentration of only 0.25 mM of SIAB is sufficient to obtain a maximum signal in the model system. In contrast, with 40% of methanol, more than 2.5 mM of SIAB is necessary (data not shown). This leads to the conclusion that the activation of Protein G with SIAB should be preferentially performed in PBS pH 7.4 with 40% of DMSO. In the case of SIA, the exceptionally high reactivity even with methanol has to be taken into consideration [53]. For SIA, a stock solution in acetonitrile seems to be preferable.

### 3.6. Crosslinking Yield of the Protein A/G IgG System

**Figure 8:**
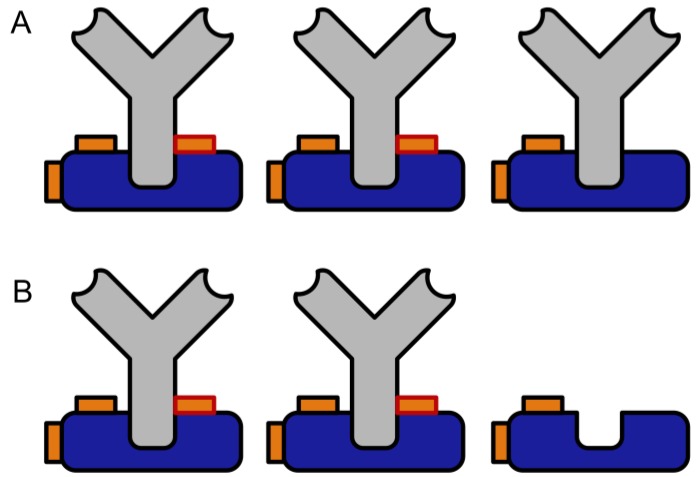
Elution test for the determination of the crosslinking yield. (**A**) Bound antibodies before the elution step and (**B**) Bound antibodies after the elution step. Any non-crosslinked antibody is lost in a subsequent washing step.

The crosslinking yield of the method was determined by an additional dissociative elution step with acidic glycine/HCl buffer, which is a proven approach to elute IgG from Protein A or G columns. Any non-crosslinked IgG should be lost during this step, leading to a loss of binding capacity. Figure 8 shows a schematic representation of this test.

**Figure 9:**
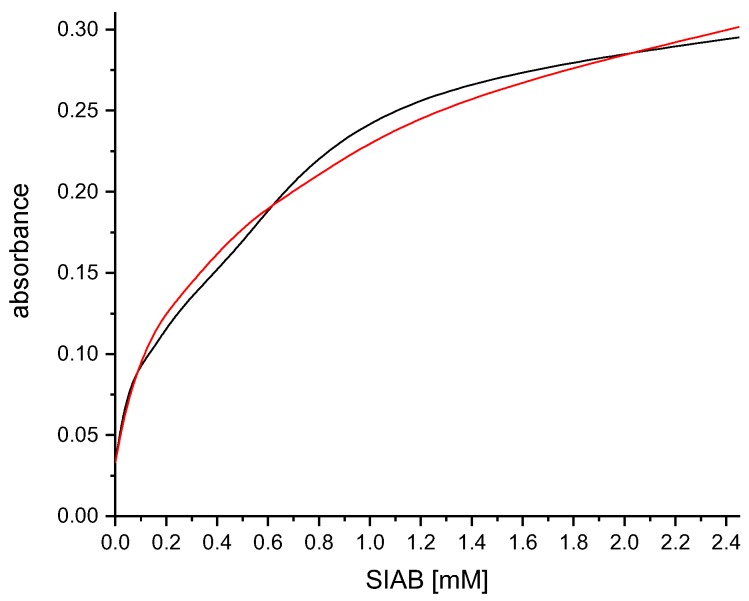
Examination of the crosslinking yield of SIAB-activated Protein G with the recombinant antibody Herceptin. Residual non-crosslinked human IgG was eluted with a glycine/HCl buffer at pH 2.2 (black: before elution, red: after elution). The crosslinking yield of the Protein G/human IgG system was apparently quantitative.

In contrast to most photochemical crosslinking protocols, the crosslinking yield with SIAB seems to be quantitative (Figure 9), at least in systems of sufficient binding strength of the protein–protein complex. However, even in the case of incomplete crosslinking, a simple pre-elution step easily gets rid of any traces of non-crosslinked antibody. This avoids leakage of antibodies into the affinity-purified sample.

### 3.7. Comparison of the Efficiency of the Traditional and the Novel Immobilization Method

**Figure 10:**
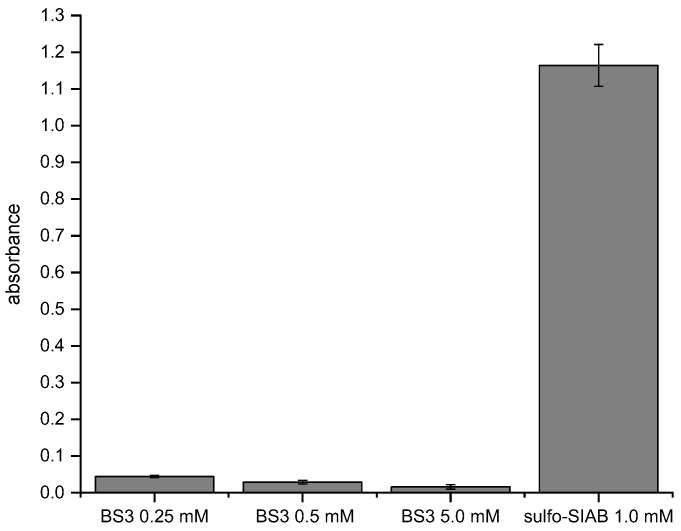
Comparison of the traditional with the novel immobilization method. BS3: Post-crosslinking of Protein G/antibody complex according to the manufacturer’s recommendation [60], sulfo-SIAB: Preactivation of Protein G with the subsequent addition of the antibody. Any non-crosslinked antibody was removed by elution buffer (glycine/HCl pH 2.2).

A traditional crosslinking protocol with the reagent bis(sulfosuccinimidyl)suberate (BS3) was compared to the proposed 2-step (preactivation) method based on sulfo-SIAB (Figure 10). It is evident that the novel method leads to a much higher signal in this model assay. Besides, the experiment shows that a higher concentration of BS3 leads to lower signals, which is most likely caused by the unwanted chemical modification of the antibody binding site as discussed. The used concentration range of BS3 is based on the manufacturer's recommendation. The concentration of sulfo-SIAB in the preactivation step was derived from our optimization experiments.

## 4. Discussion

It could be shown that some known crosslinkers can be used in a novel, 2-step protocol for oriented antibody immobilization. Up to now, Protein G/IgG or Protein A/IgG complexes have been treated with crosslinkers *after* the protein-complex had been formed, which inevitably leads to chemical changes in and near the variable region of the antibody, which is critical for selective binding and preservation of binding capacity. In our approach, Protein A or G is chemically pre-activated by an excess of homo- or heterobifunctional reagents. This did not eliminate the bioselective interaction between Protein A or G and the immunoglobulin. It can be assumed that this 2-step reaction is generally applicable for most immunoglobulins, which have some affinity to Protein A or G. A further advantage of this approach is the flexibility of the conjugation conditions, such as pH, salt concentration, additives and so on. The resulting conjugate should show no loss of binding capacity by the chemical crosslinking step since any covalent bonds are restricted to the Fc part of the antibody far away from the antigen binding site. Also, it can be assumed that no optimization of the conjugation should be required for known IgG subclasses since the regions involved in binding to Protein A or G are highly conserved. We noticed that the presence of other proteins (such as albumin) did not significantly influence the conjugation efficiency and hence, neither a pre-purification nor preconcentration of the antibody or serum is necessary. Even very raw or diluted antibody preparations might be used directly for the conjugation, which is in strong contrast to common products with pre-activated surfaces.

This selective and covalent immobilization protocol should be useful in many fields: The preparation of immunoaffinity columns, magnetic beads, the coating of nanoparticles, such as quantum dots or gold particles, the activation of glass or other slides for microarray technology, the robust coating of immunosensor surfaces, the oriented and irreversible immobilization of antibodies on microtitration plates and even homogeneous variants, such as the labeling of antibodies might be feasible. It should be stressed that in contrast to most other recent concepts, neither the production of genetically modified proteins [61] nor the introduction of synthetic amino acids is required. Most buffers, preservatives or protein additives do not limit the applicability of this approach. However, the transfer of this protocol to other biochemical binding pairs has still to be explored.

This approach might be even useful for crosslinking experiments in solution, which are highly popular in proteomics [62] and structural biology [63,64]. All experiments, which are performed with traditional thermal or photochemical crosslinkers today, could be alternatively performed with protocols analogous to those presented here. The crosslinking site might be more restricted and hence better to control.

Regarding the reaction mechanism, it seems to be evident that crosslinkers suitable for this approach need at least one active group, which does not hydrolyze or otherwise deactivate too fast. Therefore, bifunctional NHS esters [45] seem to be suboptimal. In contrast, haloacetyl-residues, such as SIA or SIAB possess a good balance between stability and reactivity towards nucleophiles. We assume that at neutral pH values, primarily histidine residues are involved in the crosslinking with haloacetyl groups, in contrast to the mechanism with glutaraldehyde, which should be dominated by lysines [52]. Due to the slow reaction, even complexes with a relatively low affinity at low protein concentrations may be accessible, in contrary to photochemical groups, which have very short reactive lifetimes and hence often low reaction yields [27] with various side reactions. Finally, we want to stress that all required reagents necessary for this novel approach are commercially available from standard suppliers. Considering the much lower signals obtained with the old method, in most applications the proposed approach should be preferred.

## Figures and Tables

**Table 1:** Compounds used for the preactivation crosslinker screening with Protein G at pH 7.4.

Crosslinker	Abbr.	Efficiency
Formaldehyde [47]	FA	-
Disuccinimidyl tartrate [48]	DST	-
Tris(hydroxymethyl) phosphine	THP	-
1-Ethyl-3-(3-dimethylaminopropyl) carbodiimide [49]/N-Hydroxysuccinimide	EDC/NHS	-
Bis(sulfosuccinimidyl)suberate [48]	BS3	+
1,3-Butadiendiepoxide	BDDE	+
Glutaraldehyde [50,51,52]	GA	++
Succinimidyl iodoacetate [53]	SIA	++
Succinimidyl (4-iodoacetyl)aminobenzoate [48]	SIAB	+++
Sulfosuccinimidyl (4-iodoacetyl)aminobenzoate [48,54]	Sulfo-SIAB	+++
